# Everyday memory failures across adulthood: Implications for the age prospective memory paradox

**DOI:** 10.1371/journal.pone.0239581

**Published:** 2020-09-25

**Authors:** Agnieszka Niedźwieńska, Józefina Sołga, Patrycja Zagaja, Magdalena Żołnierz

**Affiliations:** Applied Memory Research Laboratory, Department of Psychology, Jagiellonian University, Kraków, Poland; Nathan S Kline Institute, UNITED STATES

## Abstract

Despite the prevalence of everyday memory failures, little is known about which specific types have the strongest impact on everyday life, and whether their impact changes across adulthood. An investigation of memory failures at different ages is particularly informative to disentangle the age paradox in prospective memory, which seems to suggest that remembering to perform intended actions in everyday life improves with age. Therefore, 58 young adults, 40 middle-aged adults, and 54 elderly adults recorded their memory failures as and when they occurred during a 7-day period, and described how serious and consequential they were. Failures were coded into several subcategories of retrospective memory, prospective memory, and absent-minded lapses. It was prospective memory lapses that were overall the most common, serious and consequential ones. Young adults had substantially more prospective memory failures than the elderly and middle-aged adults who did not differ from each other. A young adult disadvantage still held up when lifestyle differences between young adults and the elderly were taken into account. Our findings support the age-related benefit previously found in naturalistic prospective memory tasks, and suggest that it is robust across various types of prospective memory tasks. The results also suggest that the benefit may result from both young adults having poor everyday prospective memory, compared to any adults of a greater age, and everyday prospective memory being spared from age-related decline between the middle and late adulthood.

## Introduction

Memory failure is a common everyday experience, with the majority of healthy adults having at least several instances per week, and many adults having several dozen. Despite the prevalence of everyday memory failures (EMFs), little is known about their consequences and the specific types that have the strongest impact on everyday life. Furthermore, knowledge is lacking as to whether EMFs are more problematic in middle and late adulthood, compared to early adulthood when memory processes are at their peak.

An investigation of EMFs at different ages is particularly informative to disentangle the age prospective memory paradox [1], which suggests that, contrary to expectations, certain types of EMFs may become less frequent with age. Prospective memory (PM) is the ability to remember to perform an action at a certain moment in the future. Even though there are significant age-related deficits on laboratory PM tasks, the elderly outperform young adults across naturalistic PM tasks carried out in everyday life [2]. Several moderators of this age-related benefit have been found [3–5], but some issues remained unresolved.

For instance, the benefit may result from lifestyle differences between the older adult participants and students, who are invariably the young adult participants in studies on naturalistic PM tasks. Compared to students, the elderly possibly have more regular patterns of living with a higher number of routine activities, which may give them more opportunities to plan the execution of PM tasks (e.g., in conjunction with routine events) [6]. Furthermore, higher routinisation among the elderly would involve a more automatic execution of well-learned activities, which may reduce the overall frequency of everyday forgetting. Nevertheless, Rendell and Craik [1] argued that the age-related benefit in naturalistic PM tasks reflects real differences in motivational and cognitive factors between young adults and the elderly, and that it cannot be explained away by lifestyle. Rendell and Thomson [6] did not find differences between elderly groups, whose occupations were either work, home duties, or retired, on naturalistic PM tasks. However, no systematic studies on the contribution of differences in the level of routine to the age benefit have been conducted.

Furthermore, the benefit may have been overestimated due to the dominant use of naturalistic (carried out in everyday life), but experimenter-generated PM tasks, e.g., remembering to contact the experimenter at designated times. The pattern of age-related differences may depend on the type and content of the PM task (e.g., regular vs one-off, related to other people vs not-related) [3, 7, 8], and therefore investigation should be extended to diverse real-life PM tasks formed by the participants themselves. When age-related effects were examined across all the intentions that participants had set up themselves, the benefit occurred in some studies [3, 4], but not in others [7]. When Schnitzspahn et al. [8] considered the different contents of self-assigned intentions, the benefit only held true for specific types of intentions. The issue of whether the age-related benefit is restricted to certain PM tasks may be effectively addressed by the diary method, in which EMFs are recorded immediately whenever they are noticed [9, 10]. This method fully captures the diversity of everyday memory tasks on which an individual can fail.

Therefore, the present study compared EMFs recorded in diaries by young, middle-aged and elderly adults. To precisely identify the types of EMFs that have the strongest impact on everyday life, and which are subject to age-related differences, we classified EMFs into categories reflecting distinct memory processes. In addition, adults at different ages were compared in terms of how serious and consequential different types of EMFs were. To the best of our knowledge, only two published studies have compared young and elderly adults on EMFs recorded as and when they occurred [11, 12]. They have produced conflicting results, with younger adults having either less EMFs [11] or more [12], compared to the elderly. None of them addressed the issue of consequences.

In contrast to previous studies in which EMFs were recorded immediately whenever they were noticed [11, 12], we included middle-aged adults in age comparisons. Including middle-aged adults is particularly informative to disentangle the age-related benefit in real-life PM. If the elderly outperform young adults, the question is whether it is young adults who are very poor on real-life PM tasks compared to any adults of a greater age, or whether it is older adults who are remarkably good compared to any adults of a lesser age. So far, only two studies compared the performance of middle-aged adults on naturalistic and real-life PM tasks with the performance of young and elderly adults [4, 13]. Both studies showed that middle-aged adults performed as well as the elderly, and better than young adults.

In addition, we analysed whether the extent to which the young and elderly adult participants had regular patterns of everyday activities, would influence the overall frequency of failures and age-related differences in EMFs. To this aim, we recruited two groups of young adults in the same age range, with one group made up of students and the other group comprised of young workers. It was based on the assumption that a full-time job made the daily life of young workers more routinised, compared to young students. We also recruited two groups of the elderly in the same age range. One group included retired older adults who were not enrolled in any educational or leisure activity program, and the other group comprised of retired older adults who participated in frequent but irregular activities organised by the University of the Third Age. It was based on the assumption that these frequent but irregular activities made the daily patterns of Third Age students less routinised, compared to the other group of the elderly.

In line with the results of previous diary studies on EMFs [9, 12, 14], we expected that overall PM lapses would be more frequent than other types of EMFs (but see [15], for a different pattern). In accordance with a meta-analysis on the age-related benefit in everyday PM [2], we expected that young adults would have more PM failures than the elderly. Based on speculations made both in the literature on EMFs [9, 16], and in the literature on the age-related benefit in naturalistic PM tasks [1, 6], we expected that lifestyle would influence the frequency of EMFs, i.e., participants with less regular patterns of everyday activities would have more failures than those with more routinised life. However, drawn on the arguments of Rendell and Craik [1], we predicted that varying the level of routine would not change the pattern of age-related differences, i.e., young adults would still have more PM failures than elderly adults. Based on studies of the age benefit in which middle-aged adults were included [4, 13], we expected middle-aged adults to have less PM failures compared to young adults, with no differences between middle-aged and elderly adults.

## Method

### Participants

A total of 152 adults, including 58 young adults (age range 19–30), 40 middle-aged adults (age range 35–55), and 54 older adults (age range 61–80), were recruited. They were volunteers from the community who responded to invitations disseminated at universities, companies, public offices, community groups, social clubs for older adults, Universities of the Third Age, as well as through a friendship network. They did not receive any remuneration for their participation. The study was approved by Psychology Research Ethics Committee at the Jagiellonian University in Kraków.

Half the young adult participants were undergraduate students from various universities, whereas the other half were young adults who were not enrolled in any educational program, but were employed and worked full-time. Similarly to the middle-aged adult participants, they were predominantly white-collar workers.

All the middle-aged adult participants were employed and worked full-time.

Half the older adult participants were members of the University of the Third Age who, on a continuous basis, attended events and activities offered by the University. The activities included academic activities (lectures and workshops) and fitness classes. The events included cultural events (e.g., attending concerts, theater performances), sightseeing tours, as well as parties and other social events. These events and activities were organised at frequent but irregular times, except for some fitness classes. Not only cultural and social events came in irregular patterns, but also academic activities were offered only when the University of the Third Age managed to invite an academic to give a lecture or workshop. Lectures usually consisted of one-off events and workshops were conducted in one go, over the course of a few days. The other half of the older adult participants were not members of Universities of the Third Age and were not involved in any formal program of education or fitness and leisure. All the older adult participants were screened for the presence of neurological or psychiatric disorders, including dementia, using the Polish version of the Mini-Mental State Examination (MMSE) [17]. None of them scored below the cut off score of 27 and all attained a maximum score on episodic memory, as measured by the recall task in the MMSE. All older adults were physically active, healthy, and living independently.

All participants had at least secondary education and lived in urban areas of southern Poland. Table 1 shows demographic details of the sample. A one-way ANOVA and a cross-tabulation table (for gender) revealed no significant differences between the three age groups in gender balance and education, *p*_s_ > 0.272. In addition, a series of independent samples t-tests revealed no significant differences between the two groups of elderly adults (Third Age students and those unenrolled in the University) in age, education, and MMSE scores, *p*_s_ > 0.115. Chi-square tests revealed no significant differences between these two groups in gender balance and the percentage of adults who were retired, p > 0.685. Finally, the two groups of young adults (students and workers) did not differ in gender balance and education, *p*_s_ > 0.165. Students were younger than workers, t(56) = -5.27, p < 0.001, d = 1.38. However, the difference in years was small (see Table 1), with the majority of participants in both groups being between 22 and 27 years old: students 86%, young workers 55%.

**Table 1:** Demographic characteristics (means and SDs) of young adults (students vs. workers), middle-aged adults and older adults (enrolled vs. unenrolled in the University of the Third Age).

	Young adults			Middle-aged adults	Older adults		
	Students	Workers	Total	Total	Enrolled in U3A	Unenrolled in U3A	Total
	n = 29	n = 29	n = 58	n = 40	n = 27	n = 27	n = 54
% women	52	52	52	55	67	63	65
Age	22.62 (1.40)	25.41 (2.49)	24.02 (2.45)	44.20 (6.29)	71.85 (3.93)	73.96 (5.61)	72.91 (4.92)
Education (years)	15.28 (1.19)	15.90 (2.06)	15.59 (1.70)	15.63 (1.97)	14.67 (2.30)	15.44 (2.24)	15.06 (2.28)
% retired					89	85	
MMSE					29.89 (0.32)	29.93 (0.27)	

*Note*. MMSE = Mini-Mental State Examination; U3A = the University of the Third Age

### Measures

#### Paper diary

The diary study was designed in accordance with the guidelines for paper and pencil diaries [18, 19], i.e., the diary had been piloted with different age groups, each entry took no more than 2–3 minutes to complete, a diary booklet was portable, and participants were thoroughly trained in how to keep a diary. Two recommended compliance-enhancing procedures were also introduced: (i) we contacted each participant during the diary keeping period to remind them how important it was to maintain the diary, and (ii) the diary instructions required participants to write down not only the time when they had a memory failure, but also the time when they recorded it.

Participants received an A5 paper diary booklet, containing 32 identical pages, one page to be completed for each EMF experienced. In addition to describing each failure and reporting when it happened and when it was recorded, participants had to answer several questions about circumstances in which the failure occurred. The following items were presented on each diary page: 1. When did you have a memory error? Or when did you realise you made an error? (Date and time); 2. When did you record it here? (Date and time); 3. Describe your memory error (free text entry); 4. What was your mood immediately before the error (a 5-point scale: 1 = very unhappy to 5 = very happy, plus don’t know); 5. How relaxed or stressed were you immediately before the error (a 5-point scale: 1 = very relaxed to 5 = very stressed, plus don’t know); 6. How serious was the memory lapse (1 = insignificant, 2 = minor, 3 = somewhat significant, 4 = significant, 5 = very significant/potentially dangerous); 7. Were there or could there have been any consequences? (free text); 8. How upset are you by the memory lapse? (1 = not at all upset, 2 = a little, 3 = somewhat, 4 = quite; 5 = very upset); 9. Describe the emotions you felt in response to your lapse, if any (free text); 10. If you later recovered from this error, describe when and how (free text). This question allowed participants to describe how they remembered what they had previously forgotten.

#### Diary compliance questionnaire

A diary compliance and feedback questionnaire was completed after the diary-keeping phase. Participants had to indicate whether they carried the diary with them every day of the study (yes/no). If the ‘no’ option was chosen, they indicated how many days they did not keep the diary with them. Participants also had to estimate what percentage, out of all the memory failures they had experienced in the 7-day period, that they were able to record. Finally, they indicated whether they thought that recording memory failures had any effect on the number of failures experienced on a 7 point-scale (1 = significantly reduced the number of failures, 4 = had no effect, 7 = significantly increased the number of failures).

### Procedure

Participants were tested individually, predominantly at the participant’s home. Two sessions were conducted a week apart. At the beginning of Session 1, participants completed the consent form, demographic items, and the MMSE. The participants were then trained for about 25 minutes on how to keep a diary of EMFs. It was stressed that people of all ages complain that they forget things they know they should remember and that these memory failures take different forms, which was followed by examples of retrospective, prospective, and absent-minded lapses. The three categories as such, were not explicitly mentioned or defined. Three examples of failures from each category were provided, and the order of category presentation was counterbalanced across participants. To ensure that participants understood the idea of EMFs, they were asked to give examples of memory lapses they had recently experienced.

Participants were then asked to record any EMFs that occurred over the next seven days, starting from waking the day after the briefing, so that only full days were recorded. They were urged to keep the diary with them at all times and to record memory failures immediately, or as soon as possible after their occurrence. Participants were informed that, on some occasions, they would notice a memory failure as soon as it happened and then they should record it, e.g., they would feel that they cannot remember the name of the friend’s wife, or they would notice that they had passed the shop which they had wanted to pop into. On other occasions, they would realise that they had a failure some time after its occurrence and then they should record it, e.g., when they opened the fridge in the evening they would realise that they had forgotten to buy milk on the way home, or when going to work in the morning they would realise that they had forgotten to lock the car the previous day.

Participants were also informed that it would not always be possible, or appropriate, to record failures, because of activities such as driving, or during meetings. If that was the case, and to minimize making retrospective entries, they were advised that if they could not complete the diary page immediately and later felt that they could not recall key characteristics, they could record them as a tick (on a grid, with rows for the appropriate day, on the inside front cover of the paper diary). Participants were also urged to record each failure they would experience, no matter how trivial or unimportant it may seem to them. Each item on the diary page was then explained. Possible answers to each question were discussed, using the examples of EMFs that had been provided by the participant.

In addition to the verbal training, participants were given written instructions on how to complete their paper diary. Written instructions were provided on the last page of the paper booklet so that participants could consult them at any time when keeping a diary. The experimenter phoned each participant once, during the first few days of keeping a diary.

In Session 2, the experimenter took the diary booklet and the participant was asked to fill in the Diary Compliance Questionnaire.

### Coding participants’ descriptions of memory failures

The analyses of diary entries in terms of the types of memory failures were based on a coding system developed by Kvavilashvili et al. [20]. They asked participants to provide the descriptions of their most recent memory failures, which were subjected to thematic content analysis. A bottom up approach was used, without any pre-existing classification scheme. This classification system is also in line with a current theoretical approach to memory that delineates several distinct memory systems [21], and specifically distinguishes working memory [22] from long-term (retrospective) memory, on the one hand, and PM from retrospective memory, on the other [23].

In the coding system, retrospective memory lapses refer to forgetting information from the past and include forgetting: names, PIN-Codes, facts, locations, items from shopping lists, as well as forgetting that actions have already been completed. PM lapses refer to forgetting to perform an intended activity at a particular point in the future and include both forgetting to do things several minutes later (e.g., to turn off the oven when the roasted dish is ready) and, in the longer term, things such as forgetting to take medication. The following example should help to clarify the distinction. If a person wants to pass on a message to a colleague when they see her at work, but this intention is not retrieved from memory at the right future moment (when they see the colleague), it is a PM lapse. If the person sees the colleague and remembers that they need to pass on a message to her, but does not remember what message they decided to pass on, it is a retrospective memory lapse (see [23] for more details about this distinction). Absent-minded lapses refer to forgetting information that is necessary for ongoing processing and action regulation that should be maintained in working memory. They include temporary loss of the content of intention (Why am I here?), doing one thing instead of another, not finishing a started sequence or temporary disorientation regarding the day, date or time. In contrast to PM failures, in which the person has to store an intention in memory for some time (there is a delay between forming an intention to do something and the opportunity to perform it), in absent-minded lapses the person forgets to perform part of the ongoing process or sequence. The coding system includes several subcategories of lapses, developed by Niedźwieńska and Kvavilashvili [10], within each broad category of retrospective, prospective and absent-minded failures (see Table 2).

**Table 2:** Number of lapses in each specific subcategory in young, middle-aged and older adults respectively.

Retrospective memory lapses	Prospective memory lapses	Absent-minded lapses
*they could not remember …*	*they forgot …*	
**Names, words** (31_a_/4_b_/22)**	about medical/private **Appointments** or somebody’s birthday (13/16/32)	**Temporary Disorientation** regarding the day, date or time (1/3/1)
**PIN-Codes, phone numbers, addresses** that had been in long-term use (12/6/2)	to take **Medication** (14/8/20)	**Omitting an action** in the sequence of actions, but not the last action (15/5/14)
**What they had been told** or that they had said something already (15/2/8)	to **Get in touch**, e.g., make a phone call, send a text message or visit (76_a_/13_b_/14_b_)***	**Not finishing** the sequence of actions: forgetting to perform the last one (11/8/21)
**Shopping item**: forgot one or more items when shopping (21/10/30)	to **Pass on** a message or return something when they had seen somebody (42_a_/4_b_/5_b_)***	**Action swap**: doing another thing instead of an intended action (4/4/7)
**Directions to destination** (4/1/1)	to **Go to buy**/order/collect something (58_a_/26/20_b_)*	**Forgetting why they came** to a certain location at home or what they wanted to do (43_a_/5_b_/8_b_)***
**Where they had hidden**/put something at home, e.g., the wedding invitation, a spare battery charger (15/14/6)	to **Do something after a certain period** of time, e.g., burnt meat when cooking (18/6/26)	**Leaving behind** something that was in sight, e.g., leaving the bought items at the counter (8/4/4)
**Plans:** what will be happening that day, e.g., food is delivered (in contrast to things they need to remember to do themselves) or some content of their intention, e.g., the time of the meeting (22/12/5)	to **Take something extra from home** that was needed during that day, e.g., extra clothes, an umbrella, fitness stuff (in contrast to things they always take with them when leaving home) (107_a_/27_b_/25_b_)***	**Forgetting to take usual things from home**, i.e., things that they always take with them when leaving home, e.g., a wallet, house keys (13/7/11)
**Their actions:** thought they had not done something, but they had or they did not know whether they had done something or not (12/6/6)	to **Complete a one-off activity**, e.g., to arrange an appointment or withdraw money from cash machine (139_a_/51_b_/48_b_)***	**Misplacing things** that are in constant use at home, e.g., a mobile phone and have their usual location, e.g., house keys (8/0/9)
	about **Regular duties** at home, e.g., feed the pet, brush the teeth (12/13/5)	

*Note*. Asterisks indicate significant group differences:

*p<0.05.

**p<0.01.

***p<0.001; values with different subscripts differed at the 0.0167 level

Using the coding system, all diary entries were coded by two coders (independently and being blind to both participant group and the hypotheses of the study). Inter-rater reliability between the coders was from strong to almost perfect, with Cohen’s weighted κ_s_ from 0.87 (*SE* = 0.02) to 0.96 (*SE* = 0.01) [24], and disagreements were resolved by discussion. Out of 1327 diary entries that were provided by the participants, only eight entries were found, by both coders, too vague for coding and were excluded from the further analyses.

## Results

For all statistical tests, the rejection level was set at .05 (unless otherwise specified). The effect size, as measured by partial eta-squared (η^2^_p_), was defined as 0.01, 0.06, and 0.16 for small, medium and large effects respectively [25]. As recommended for multiple tests of simple main effects [26], the Bonferroni correction for multiple comparisons were used, i.e., alphas were divided by the number of simple main effects tested for a given factor. For the non-parametric Mann-Whitney tests, used in pair-wise comparisons on the number of lapses from each subcategory within each broad category of prospective, retrospective and absent-minded failures (Table 2), the Bonferroni correction was also applied [27].

### Compliance and the effect of keeping a diary on the number of failures

A one-way ANOVA (when comparing the three age groups) and t-tests for independent samples (when comparing the two groups of young adults or the two groups of elderly adults) did not reveal group differences in the number of days during which participants kept the diary with them, *p*_s_ > 0.171 (see Table 3). The majority of participants in each group kept the diary with them every day for the entire 7-day period (between 75% and 93%), with no differences between the groups, *p*_s_ > 0.125. The minimum number of days during which participants kept a diary was 5 in each group. In addition, the groups did not differ in the percentage of total number of failures experienced that participants felt they had reported in the diary, *p*_s_ > 0.100. Furthermore, the groups did not differ in the effect that the process of recording failures had on the number of failures experienced, i.e., whether participants thought it significantly reduced or increased the number of lapses, ps > 0.300 (see Table 3). All but 6 participants claimed that keeping a diary either reduced the number of failures experienced or did not have any effect on the number of failures.

**Table 3:** Mean (standard deviation) days of keeping a diary, percentage of failures recorded, and effect of keeping a diary on the number of failures in young adults (students vs. workers), middle-aged adults, and older adults (enrolled vs. unenrolled in University of the Third Age).

	Young adults			Middle-aged adults	Older adults		
	Students	Workers	Total	Total	Enrolled in U3A	Un-enrolled	Total
	n = 29	n = 29	n = 58	n = 40	n = 27	n = 27	n = 54
Days of keeping a diary	6.90 (0.41)	6.72 (0.59)	6.81 (0.51)	6.60 (0.74)	6.70 (0.61)	6.89 (0.42)	6.80 (0.53)
Percentage of failures recorded	84.12 (14.16)	76.52 (20.08)	80.32 (17.64)	79.90 (17.45)	86.41 (16.45)	81.19 (12.26)	83.80 (14.61)
Effect on the number of failures	3.17 (1.20)	3.28 (1.07)	3.22 (1.13)	2.88 (1.38)	3.26 (1.16)	3.15 (1.03)	3.20 (1.09)

*Note*. U3A = the University of the Third Age; Effect of keeping a diary on the number of lapses (1 = *significantly reduced the number*, 4 = *had no effect*, 7 = *significantly increased the number*)

Turning to the main findings, a total of 1319 EMFs with a mean of 8.68 (*SD* = 5.12; range 2–25) failures per person were recorded. Of these, 838 (64%) were PM failures which were recorded, in line with our expectations, significantly more often than retrospective memory failures (267; 20%) and absent-minded failures (214; 16%), *p*_s_ < 0.001.

### The frequency of memory failures in three age groups

**Fig 1:**
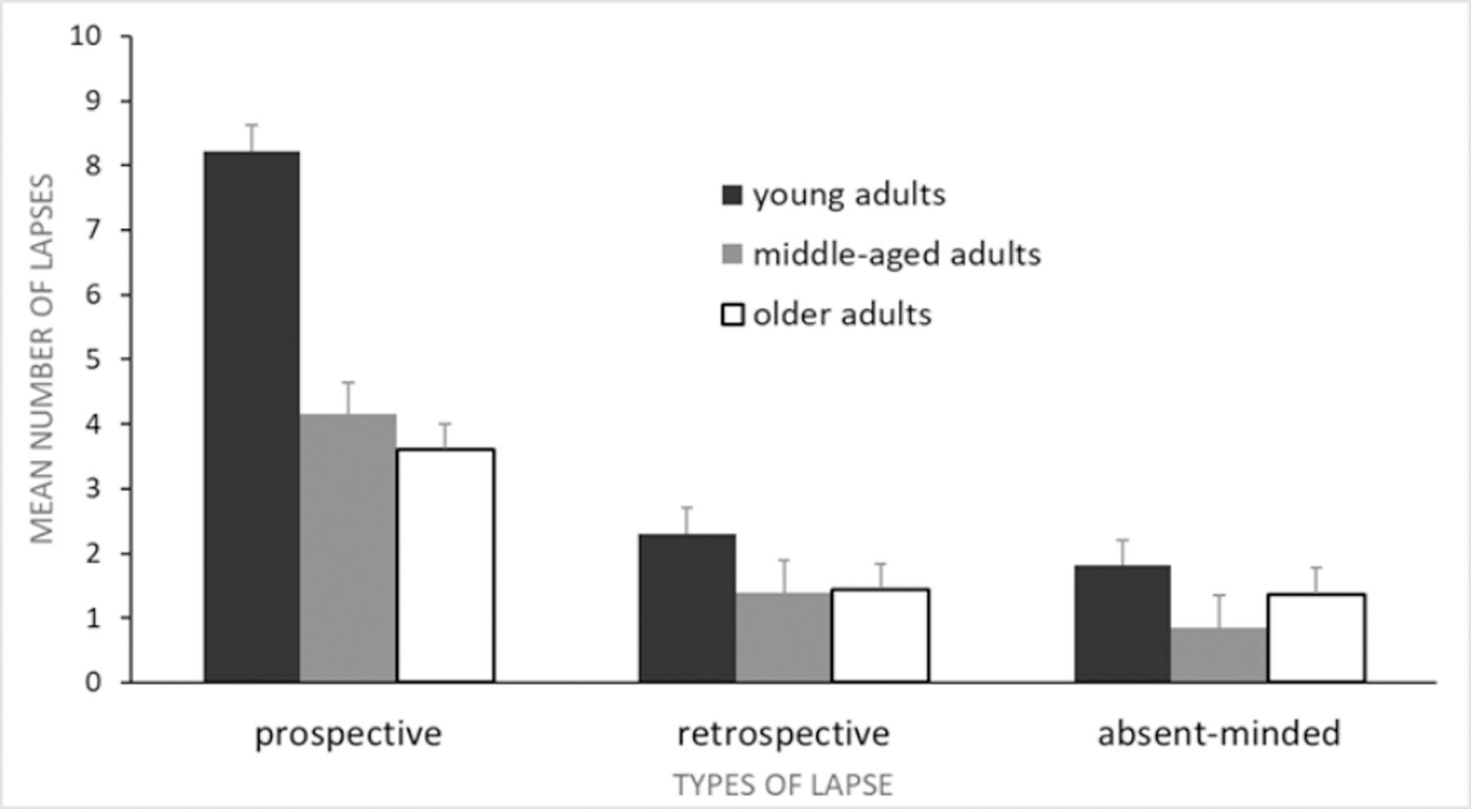
Mean number of memory lapses as a function of type of lapse (prospective, retrospective, absent-minded) and group (young adults, middle-aged adults, older adults). Error bars represent 1 SE of the mean.

The number of EMFs in each category were entered into a 3 (group: young adults, middle-age adults, elderly adults) × 3 (type of lapse: retrospective, prospective, absent-minded) mixed ANOVA with repeated measures on the second factor (see Table 4). The main effect of group was significant, *F*(2,149) = 34.86, *p* < 0.001, η^2^_p_ = 0.32. The main effect of type of lapse was also significant, *F*(1.607, 239.488) = 119.45, *p* < 0.001, η^2^_p_ = .45. However, these main effects were qualified by a significant group by type of lapse interaction, *F*(3.215, 239.488) = 13.56, *p* < 0.001, η^2^_p_ = 0.15 (see Fig 1).

**Table 4:** Mean (standard deviation) number of memory lapses as a function of type of lapse (prospective, retrospective, absent-minded) and group (young adults, middle-aged adults, older adults).

	Young adults	Middle-aged adults	Older adults
	n = 58	n = 40	n = 54
Prospective	8.22_a_^f^ (4.63)	4.15_a_^g^ (2.38)	3.61_a_^g^ (2.40)
Retrospective	2.31_b_ (2.18)	1.40_b_ (1.47)	1.44_b_ (1.51)
Absent-minded	1.81_b_ (2.25)	0.85_b_ (0.92)	1.37_b_ (1.65)

Within a column, means with different subscripts differed in HSD Tukey tests; Within a row, means with different superscripts differed in HSD Tukey tests.

Tests of simple main effects, with alpha level corrected to 0.0167, showed that the groups significantly differed in the frequency of PM lapses, *F*(2, 149) = 29.70, *p* < 0.001, η^2^_p_ = 0.29, and retrospective memory lapses, *F*(2, 149) = 4.41, *p* = 0.014, η^2^_p_ = .06, but they did not differ in the frequency of absent-minded lapses, *F*(2, 149) = 3.50, *p* = 0.033, η^2^_p_ = 0.05. Post hoc tests revealed that young adults reported more PM lapses than middle-aged adults and elderly adults, *p*_s_ < 0.001, which did not differ from each other, *p* = 0.981. Although a test of simple main effect showed a significant difference between the groups in the frequency of retrospective memory lapses, post hoc tests did not reveal any significant differences in pair-wise comparisons for this type of lapse, *p*_s_ > 0.636.

An additional set of tests of simple main effects, with alpha level corrected to 0.0167, showed that the main effect of type of lapse was significant in young adults, *F*(2,148) = 84.57, *p* < 0.001, η^2^_p_ = 0.53, middle-aged adults, *F*(2,148) = 15.13, *p* < 0.001, η^2^_p_ = 0.17, and elderly adults, *F*(2,148) = 9.85, *p* < 0.001, η^2^_p_ = 0.12. Post hoc tests revealed that the number of PM lapses was significantly higher than the retrospective memory lapses and absent-minded lapses for young adults, *p*_s_ < 0.001, middle-aged adults, *p*_s_ < 0.001, and older adults, *p*_s_ < 0.001. The number of the retrospective memory lapses and absent-minded lapses did not differ from each other in any of the groups, *p*_s_ > 0.974.

### Lifestyle and the differences between young adults and the elderly in the frequency of memory failures

The number of EMFs in each category were entered into a 2 (group: young adults, the elderly) × 2 (lifestyle: more routinised/less routinised) × 3 (type of lapse: retrospective, prospective, absent-minded) mixed ANOVA with repeated measures on the third factor. The main effect of lifestyle did not reach the level of statistical significance, *F*(1,108) = 3.37, *p* = 0.069, η^2^_p_ = 0.03. Lifestyle did not interact with age, *F*(1,108) = 0.03, *p* = 0.887, η^2^_p_ = 0.00, type of lapse, *F*(1.609, 173.733) = 1.22, *p* = 0.296, η^2^_p_ = 0.01, and age and types of lapse, *F*(1.609, 173.733) = 0.02, *p* = 0.980, η^2^_p_ = 0.00. The pattern of results for the two remaining factors, i.e., group and type of lapse, was exactly the same as when the three age groups were compared (see the analyses above). The main effect of group was significant, *F*(1,108) = 46.43, *p* < 0.001, η^2^_p_ = 0.30. The main effect of type of lapse was also significant, *F*(1.609, 173.733) = 91.35, *p* < 0.001, η^2^_p_ = .46. These main effects were qualified by a significant group by type of lapse interaction, *F*(1.609, 173.733) = 20.57, *p* < 0.001, η^2^_p_ = 0.16. Tests of simple main effects, with alpha level corrected to 0.0167, showed that the groups significantly differed in the frequency of PM lapses, *F*(1, 108) = 43.25, *p* < 0.001, η^2^_p_ = 0.29, with young adults having more PM lapses than the elderly. The groups did not differ in the frequency of retrospective memory lapses, *F*(1, 108) = 5.80, *p* = 0.018, η^2^_p_ = 0.05, and absent-minded lapses, *F*(1, 108) = 1.35, *p* = 0.247, η^2^_p_ = 0.01.

All further analyses were conducted on the three age groups.

### Seriousness and disturbance ratings

We performed a series of ANOVAs on mean ratings of how serious the failure was, and how upset the participant was by the failure in each category of lapses. The mean ratings were entered into a 3 (group: young adults, middle-aged adults, elderly adults) ×3 (type of lapse: retrospective, prospective, absent-minded) mixed ANOVAs. The main effect of type of lapse on the seriousness ratings was significant, *F*(1.640, 108.237) = 7.60, *p* < 0.001, η^2^_p_ = 0.10. Post hoc tests revealed that participants considered PM lapses more serious (*M* = 2.40, *SD* = 0.67) than retrospective memory lapses (*M* = 2.06, *SD* = 0.84) and absent-minded lapses (*M* = 1.98, *SD* = 0.86), *p*_s_ < 0.01, which did not differ from each other, *p* = 0.745. The main effect of type of lapse on how upset the participant was by the failure was also significant, *F*(1.879, 124.038) = 4.83, *p* = 0.010, η^2^_p_ = 0.07. Post hoc tests revealed that participants were more upset by PM lapses (*M* = 2.52, *SD* = 0.82) compared to absent-minded lapses (*M* = 2.06, *SD* = 1.02), *p* = 0.005. The ratings for retrospective memory lapses (*M* = 2.21, *SD* = 1.01) did not differ from the ratings for PM lapses and absent-minded lapses, *p*_s_ > 0.069.

### Consequences

Participants reported consequences for 48% of EMFs. The numbers of those lapses for which consequences were reported were entered into a 3 (group: young adults, middle-aged adults, elderly adults)×3 (type of lapse: retrospective, prospective, absent-minded) mixed ANOVA (see Table 5). The main effect of group was significant, *F*(2,68) = 4.31, *p* = 0.017, η^2^_p_ = 0.11. The main effect of type of lapse was also significant, *F*(1.482, 100.757) = 44.38, *p* < 0.001, η^2^_p_ = .40. However, these main effects were qualified by a significant group by type of lapse interaction, *F*(2.963, 100.757) = 5.72, *p* < 0.001, η^2^_p_ = .14. Tests of simple main effects, with alpha level corrected to 0.0167, showed that the main effect of type of lapse was significant in young adults, *F*(2,67) = 27.71, *p* < 0.001, η^2^_p_ = 0.45, and middle-aged adults *F*(2,67) = 10.00, *p* < 0.001, η^2^_p_ = 0.23, but not in older adults *F*(2,67) = 1.39, *p* = 0.256, η^2^_p_ = 0.04. Post hoc tests revealed that, for both young and middle-aged adults, the number of lapses with consequences was significantly higher for PM lapses than retrospective memory lapses and absent-minded lapses, *p*_s_ < 0.001, which did not differ from each other, *p* > 0.631.

**Table 5:** Mean (standard deviation) number of failures for which consequences were reported as a function of type of lapse (prospective, retrospective, absent-minded) and group (young adults, middle-aged adults, older adults).

	Young adults	Middle-aged adults	Older adults
	n = 30	n = 18	n = 21
Prospective	4.43_a_ (3.62)	3.37_a_ (2.48)	1.55 (1.63)
Retrospective	1.10_b_ (1.19)	1.00_b_ (0.88)	0.91 (1.93)
Absent-minded	0.97_b_ (1.03)	0.68_b_ (0.95)	0.64 (0.85)

*Note*. The analyses were conducted on much smaller sample because many participants did not have lapses that belonged to all three types of lapses and they were excluded from the analyses; Within a column, means with different subscripts differed in HSD Tukey tests.

The consequences of PM lapses involved: (i) wasted time, e.g., participants forgot to stop at a store on the way home and needed to do shopping later, (ii) being late for work or meetings because they needed to go back home for the forgotten things, (iii) wasted money, e.g., fines for forgetting to return books on time or to take the mandatory documents when driving, (iv) destroyed things, e.g., a favourite piece of clothing destroyed because they forgot to lower the water temperature or to complete hand washing, (v) physical discomfort, e.g., getting cold or wet because they forgot to take an umbrella or warm clothes from home, (vi) delays and difficulties in arranging a subsequent appointment when participants forgot to attend the previous one, (vii) some danger for health, e.g., forgetting to take medication that had to be taken on time such as antibiotics or insulin.

Interestingly, participants described a whole range of emotional and social consequences of PM failures such as their own embarrassment and the negative responses of other people. Negative responses included others: (i) being sad and disappointed because participants forgot about their birthdays or anniversaries, (ii) got offended because of failing to get in touch with them, (iii) being angry because participants forgot, for example, to give them a lift or to pass on an important message to them (iv) losing confidence in the participant because they forgot to do what they promised to do or to return something.

## Discussion

Despite the prevalence of EMFs, little is known about which specific types have the strongest impact on daily life, and whether their impact changes across adulthood. Therefore, we compared, for the first time, young, middle-aged and elderly adults on various types of EMFs as well as on how serious and consequential they were.

Several important findings emerged from these comparisons. First, in line with our predictions, PM failures were the prevalent form of EMFs reported in all age groups. It was also PM failures that were experienced as the most serious lapses and, for young and middle-aged adults, they were the prevalent form of EMFs with noticeable consequences. These latter findings confirm earlier speculations [28, 29] that PM failures may be the most problematic of all failures as they particularly undermine peoples’ ability to lead safe and effective lives. However, our findings further add to this argument by showing that PM failures have also significant consequences of a social and emotional nature, including losses in the social perception of an individual’s reliability in both private and work-related contexts.

Second, and again in line with our expectations, young adults reported experiencing substantially more PM lapses than the elderly. This finding supports the age-related benefit that has previously been found in naturalistic PM tasks, both experimenter-generated and formed by the participants themselves [2], and shows that the phenomenon holds across different methods of analysing everyday PM. Importantly, a young adult disadvantage still held up when lifestyle differences between young adults and the elderly were taken into account. Specifically, the pattern was observed both when the groups were similar in lifestyle to those used in previous studies on the age benefit in PM (students vs mostly retired older adults), and when young workers with relatively regular daily patterns were compared with mostly retired older adults, whose life became less regular due to the University of the Third Age. This finding speaks against the criticism that has been repeatedly raised against the age-related benefit in everyday PM [30], i.e. that the benefit can be explained away by age-related differences in lifestyle.

Third, we found that young adults, compared to the elderly, recorded more difficulties on a whole range of PM tasks (see Table 2). These included tasks involving other people (remembering to get in touch and pass on something) and not-related to other people (remembering to take something from home and go shopping). Several researchers have suggested that the age-related benefit may hold true only for a certain type of intentions [7, 8]. For instance, Schnitzspahn et al. [8] found a young adult disadvantage only for the health and social intentions. They attributed this to the elderly having more experience with health-related tasks and placing more importance on social intentions, compared to young adults. In contrast to that, our findings support the global robustness of the age benefit by showing that a young adult disadvantage was not restricted to any specific type of PM task.

Furthermore, the present investigation puts age-related differences in a lifespan context by including middle-aged adults, and showing that they had less PM lapses than young adults, and no less than the elderly. This pattern is in line with our expectations and suggests that the age-benefit may result from two effects: (i) young adults being remarkably poor in real-life PM compared to any adults of a greater age, and (ii) real-life PM being spared from age-related decline between the middle and late adulthood. The latter effect accords well with the results of a study by Park et al. [31], in which it was middle-aged adults rather than older adults who were more at risk of having PM failures related to taking medication. The absence of a decline between the middle and late adulthood may not be just a benefit of accumulated experience, but also a specific adjustment of everyday behaviour in the face of experienced memory failures of consequence. Dixon et al. [32] argue that older adults, facing mild age-related cognitive decline, begin to adjust by relying more on compensatory strategies that include investing more time and effort into everyday remembering. Furthermore, Park et al. [31] observed that older adults, compared to middle-aged adults, engaged in more effective planning of their medication schedules.

Contrary to predictions, the lifestyle factor did not significantly affect the frequency of EMFs among young adults and the elderly. The mean number of EMFs among the young and elderly adults with less regular daily patterns was numerically higher (10.29) compared to that among the young and elderly adults with more routinised patterns (8.70), but the effect did not reach the level of statistical significance (p = .069). It may be that young adults overall do not make enough use of planning to avoid memory failures, even when their more routinised life allows them to do so, as is the case with young workers. On the other hand, older adults overall may develop strategies to minimise memory failures (e.g., detailed planning and high temporal organisation) to such a high extent, that they work even when daily patterns become less regular, as is the case with Third Age students. However, it may also be that the two recruited groups of the elderly did not differ as much as we assumed they did. We did not know much about the lifestyle of the older adults who were not enrolled in any educational or leisure activity program. It may be that a significant number of them organised a rich environment for themselves and were engaged in frequent social and physical activities that occurred in irregular patterns. If it was the case, it would be less likely to observe the effect of lifestyle on the frequency of EMFs and the pattern of differences between the young and elderly adults. Future studies may address this issue by using self-reported lifestyle data.

It may be argued that the elderly reported fewer PM lapses because of a retrospective memory problem, i.e., they did not remember that they had experienced a failure. Several arguments speak against this interpretation. First, we used the diary method in which EMFs are recorded immediately whenever they are noticed which reduces the demands placed on memory to recall them [18]. Participants did not need to store in memory whether they had completed PM tasks or not because they were making the diary entry at the moment when they realised that they had intended to do something but had forgotten. For example, they could make an entry when they opened the handbag and saw the letter which they had forgotten to post during the day or when they were thinking about an upcoming social meeting and realised that they had forgotten to inform the friend about the meeting. For this diary method the biggest challenge is not to remember that you had a lapse but to remember to carry a diary with you at all times so that you are able to make an entry as soon as you realise that you had a lapse. We controlled for the number of days during which participants remembered to keep a diary-booklet with them, and found that age groups did not differ in this respect, with no participants keeping a diary for less than 5 days. Furthermore, if fewer failures recorded by the elderly resulted from difficulties in remembering them, one would expect the elderly to recall fewer failures for both of the other two broad categories of lapses as well (the retrospective memory and absent-minded failures), which was not the case.

The fact that age-related differences were specific to PM failures also speaks against the argument that they were simply due to age-related differences in the number of opportunities to commit failures, e.g., due to the elderly having less everyday tasks to perform than young adults. With less actions to carry out, the person has less opportunities to forget not only to perform the intended action at a certain moment, but also, for example, to forget to finish the action (absent-minded lapse) or forget that they have performed the action (retrospective memory lapse). Furthermore, middle-aged adults surely did not have less opportunities to commit PM failures than young adults.

In conclusion, the results demonstrate the robustness of age-related differences in the frequency with which adults failed to remember intended actions at a particular time, and support the age-related benefit previously found in this type of memory task.

## References

[1] Rendell PG, Craik FI. Virtual week and actual week: Age-related differences in prospective memory. Appl Cogn Psychol. 2000;14: 43–62. 10.1002/acp.770

[2] Henry JD, MacLeod MS, Phillips LH, Crawford JR. A meta-analytic review of prospective memory and aging. Psychol Aging. 2004;19: 27–39. 10.1037/0882-7974.19.1.27 15065929

[3] Ihle A, Schnitzspahn K, Rendell PG, Luong C, Kliegel M. Age benefits in everyday prospective memory: The influence of personal task importance, use of reminders and everyday stress. Neuropsychol Dev Cogn B Aging Neuropsychol Cogn. 2012;19: 84–101. 10.1080/13825585.2011.629288 22136429

[4] Niedźwieńska A, Janik B, Jarczyńska A. Age-related differences in everyday prospective memory tasks: The role of planning and personal importance. Int J Psychol. 2013;48: 1291–1302. 10.1080/00207594.2012.752097 23305040

[5] Schnitzspahn K, Ihle A, Henry JD, Rendell PG, Kliegel M. The age-prospective memory-paradox: An exploration of possible mechanisms. Int Psychogeriatr. 2011;23: 583–592. 10.1017/S1041610210001651 20843394

[6] Rendell PG, Thomson DM. Aging and prospective memory: Differences between naturalistic and laboratory tasks. J Gerontol B Psychol Sci Soc Sci, 1999;54B: 256–269. 10.1093/geronb/54B.4.P256 12382595

[7] Schnitzspahn KM, Kvavilashvili L, Altgassen M. Redefining the pattern of age-prospective memory-paradox: New insights on age effects in lab-based, naturalistic, and self-assigned tasks. Psychol Res. 2018; 10.1007/s00426-018-1140-2 30588544 PMC7271051

[8] Schnitzspahn KM, Scholz U, Ballhausen N, Hering A, Ihle A, Lagner P, et al Age differences in prospective memory for everyday life intentions: A diary approach. Memory. 2016;24: 444–454. 10.1080/09658211.2015.1018276 25750997

[9] Terry WS. Everyday forgetting: Data from a diary study. Psychol Rep. 1988;62: 299–303. 10.2466/pr0.1988.62.1.299

[10] Niedźwieńska A, Kvavilashvili L. Everyday memory failures in older adults with amnestic mild cognitive impairment. J Alzheimers Dis. 2019;70: 1–19. 10.3233/JAD-190259 31177225

[11] Cavanaugh JC, Grady JG, Perlmutter M. Forgetting and use of memory aids in 20 to 70 year olds everyday life. Int J Aging Hum Dev. 1983;17: 113–122. 10.2190/H7L2-K3XK-H32K-VW89 6671811

[12] Haas M, Zuber S, Kliegel M, Ballhausen N. Prospective memory errors in everyday life: Does instruction matter? Memory, 2020;28: 196–203. 10.1080/09658211.2019.1707227 31893967

[13] Niedźwieńska A, Barzykowski K. The age prospective memory paradox within the same sample in time-based and event-based tasks. Neuropsychol Dev Cogn B Aging Neuropsychol Cogn. 2012;19: 58–83. 10.1080/13825585.2011.628374 22112250

[14] Crovitz HF, Daniel WF. Measurements of everyday memory: Toward the prevention of forgetting. Bull Psychon Soc. 1984;22: 413–414. 10.3758/BF03333861

[15] Unsworth N, Brewer GA, Spillers GJ. Variation in cognitive failures: An individual differences investigation of everyday attention and memory failures. J Mem Lang. 2012;67: 1–16. 10.1016/j.jml.2011.12.005 22468805

[16] Sunderland A, Watts K, Baddeley AD, Harris JE. Subjective memory assessment and test performance in elderly adults. J Gerontol. 1986;41: 376–384. 10.1093/geronj/41.3.376 3700988

[17] Folstein M, Folstein S, Fanjiang G. Krótka Skala Oceny Stanu Umysłowego (MMSE). Warsaw, Poland: Pracownia Testów Psychologicznych; 2009.

[18] Bolger N, Davis A, Rafaeli E. Diary methods: Capturing life as it is lived. Annu Rev Psychol. 2003;54: 579–616. 10.1146/annurev.psych.54.101601.145030 12499517

[19] Stone AA, Schiffman S. Capturing momentary, self-report data: A proposal for reporting guidelines. Ann Behav Med. 2002;24: 236–243. 10.1207/S15324796ABM2403_09 12173681

[20] Kvavilashvili L, Kornbrot DE, Mash V, Cockburn J, Milne A. Differential effects of age on prospective and retrospective memory tasks in young, young-old and old-old adults. Memory. 2009;17: 180–196. 10.1080/09658210802194366 18608976

[21] Schacter DL, Wagner AD, Buckner RL. Memory systems of 1999 In: Tulving E, Craik FIM editors. The Oxford handbook of memory. Oxford: Oxford University Press; 2000 pp. 627–643.

[22] Baddeley AD. Is working memory still working. Eur Psychol. 2002;7: 85–97. 10.1027//1016-9040.7.2.85

[23] Einstein GO, McDaniel MA. Prospective memory: Multiple retrieval processes. Curr Dir Psychol Sci. 2005;14: 286–290. 10.1111/j.0963-7214.2005.00382.x

[24] McHugh ML. Interrater reliability: The Kappa statistics. Biochem Med. 2012;22: 276–282. PMC3900052 23092060

[25] Cohen J. Statistical power analysis for the behavioural sciences. 2nd ed. Hillsdale, NJ: Lawrence Erlbaum Associates; 1988.

[26] Pedhazur EJ, Schmelkin LP. Measurement, design, and analysis: An integrated approach. Hillsdale NJ: Lawrence Erlbaum Associates; 1991.

[27] Dmitrienko A, Koch GG. Analysis of clinical trials using SAS: A practical guide. 2nd ed. Cary, N.C.: SAS Institute; 2017.

[28] McDaniel MA, Einstein GO. Prospective memory: An overview and synthesis of an emerging field. Thousand Oaks, CA: Sage Publications; 2007.

[29] Zimmermann TD, Meier B. The rise and decline of prospective memory performance across the life span. Q J Exp Psychol. 2006;59: 2040–2046. 10.1080/17470210600917835 17095485

[30] Phillips LH, Henry JD, Martin M. Adult aging and prospective memory: The importance of ecological validity In: Kliegel M, Daniel MA, Einstein GO, editors. Prospective memory: Cognitive, neuroscience, developmental, and applied perspectives. New York, NY: Lawrence Erlbaum Associates; 2008 pp. 161–185.

[31] Park DC, Hertzog C, Leventhal H, Morrell RW, Leventhal E, Birchmore D, et al Medication adherence in rheumatoid arthritis patients: Older is wiser. J Am Geriatr Soc. 1999;47: 172–183. 10.1111/j.1532-5415.1999.tb04575.x 9988288

[32] Dixon RA, de Frias CM, Bäckman L. Characteristics of self-reported memory compensation in older adults. J Clin Exp Neuropsychol. 2001;23: 650–661. 10.1076/jcen.23.5.650.1242 11778642

